# Detection and Digital Resolution Counting of Nanoparticles with Optical Resonators and Applications in Biosensing

**DOI:** 10.3390/chemosensors6020013

**Published:** 2018-03-29

**Authors:** Miguel Ángel Aguirre, Kenneth D. Long, Nantao Li, Sello Lebohang Manoto, Brian T. Cunningham

**Affiliations:** 1 Department of Analytical Chemistry and Food Science and University Institute of Materials, Faculty of Science, University of Alicante, P.O. Box 99, 03080 Alicante, Spain;; 2 Department of Bioengineering, University of Illinois at Urbana-Champaign, Urbana, IL 61801, USA;; 3 Department of Electrical and Computer Engineering, University of Illinois at Urbana-Champaign, Urbana, IL 61801, USA;; 4 Biophotonics, National Laser Centre, Council for Scientific and Industrial Research, P.O. Box 395, Pretoria 0001, South Africa;

**Keywords:** photonic crystal cavities, biosensors, nanoparticles, whispering gallery mode, reflection interference

## Abstract

The interaction between nanoparticles and the electromagnetic fields associated with optical nanostructures enables sensing with single-nanoparticle limits of detection and digital resolution counting of captured nanoparticles through their intrinsic dielectric permittivity, absorption, and scattering. This paper will review the fundamental sensing methods, device structures, and detection instruments that have demonstrated the capability to observe the binding and interaction of nanoparticles at the single-unit level, where the nanoparticles are comprised of biomaterial (in the case of a virus or liposome), metal (plasmonic and magnetic nanomaterials), or inorganic dielectric material (such as TiO_2_ or SiN). We classify sensing approaches based upon their ability to observe single-nanoparticle attachment/detachment events that occur in a specific location, versus approaches that are capable of generating images of nanoparticle attachment on a nanostructured surface. We describe applications that include study of biomolecular interactions, viral load monitoring, and enzyme-free detection of biomolecules in a test sample in the context of in vitro diagnostics.

## 1. Introduction

High resolution microscopy technologies such as electron beam microscopy and confocal fluorescence microscopy, including super-resolution methods (structured illumination microscopy (SIM), stimulated emission depletion microscopy (STED), photo-activated localization microscopy (PALM), and stochastic optical reconstruction microscopy (STORM)) that enable observation of fluorophore-tagged features with sizes below the diffraction limit of light offer the ability to observe nanometer-scale objects [1–3]. While super-resolution microscopies are powerful and indispensable research tools, they also require large and costly instrumentation, and thus there is a need to develop methods that are capable of simply observing the presence of a biological analyte, counting analytes with single-unit precision, or performing kinetic measurements of a biomolecular interaction without generating a high-resolution image of the proteins, viruses, or nucleic acids being studied. A further limitation of high resolution microscopy methods is their extremely small field of view, which is insufficient for observing arrays of sensors or biomolecular binding events that may be distributed over many square millimeters of surface area. Therefore, there are compelling needs for technologies that can perform detection and visualization of individual nanometer-scale objects without necessarily obtaining high resolution images of them. Such technologies may be more applicable than high resolution microscopy for applications such as point-of-care diagnostics, and as instruments to study fundamental biological processes at the unit level, in which the analytes interact with each other in a liquid environment.

There has been enormous recent progress in the development of nanoparticles that have physical dimensions that are on the same size scale as biomolecules and viruses, opening the way to tagging biomolecules or viruses with nearly 1-to-1 stoichiometry. Nanoparticles (NP) prepared from dielectric [4], semiconductor [5], metal [6–9], and magnetic [10,11] materials have recently become important elements of biosensor technology due to their ability to prepare their surfaces with ligands that enable them to recognize specific target molecules, and their ability to interact with electromagnetic fields in useful ways. Magnetic NPs can be used to facilitate particle manipulation while at the same time providing a mass amplification tag for acoustic biosensors [12]. Likewise, metallic NPs, comprised of silver or gold, couple with external illumination sources to generate surface plasmons, which are used to enhance local electric fields on the NP surface [13,14]. While many biosensing approaches are capable of sensing the adsorption of large numbers of NPs [15], several approaches are capable of detecting the presence of a single NP, only if the particle is adsorbed to a specific active location [16–21]. For these approaches, the majority of NPs that unfortunately land in an inactive region, remain undetected. Due to the difficulty of directing analytes to precise locations on a substrate surface where a biosensor has sensitivity, an effective approach to overcoming this limitation is to utilize a biosensor surface in which the entire surface area is active as a sensor. Through the use of an imaging detection approach, the adsorption of analytes upon any region within the field of view may be measured. Imaging-based biodetection utilizing optical sensors has been demonstrated using surface plasmon resonance [22–24], photonic crystal (PC) biosensors [25–29], and dielectric thin film interference sensors [30–33]. In these approaches, NPs, may be detected with the potential to observe the attachment of individual targets. Dark field microscopy is a useful tool for NP sensing, but with contrast that decreases as 1/r^6^ of the NP [9,34], and is not able to discriminate scattering centers that are not nanoparticles [8]. Contrast for sensing NPs can be enhanced when their absorption spectra can be coupled to dielectric high quality-factor (Q-factor) resonators [35–37] or moderate Q-factor nanostructured surfaces [38].

Recently, ultrasensitive assay technologies with “digital” analyte precision have been introduced, and summarized in reviews [39,40]. For example, the Simoa^™^ system by Quanterix [41], uses an enzymatically amplified fluorescent reporter attached to antibody-functionalized micron-scale magnetic beads isolated in 50-fl reaction chambers to achieve non-multiplexed fM-scale detection limits with a more complex protocol than enzyme-linked immunosorbant ELISA. Likewise, the Erenna immunoassay technology offered by Singulex claims 1 fM detection limits using functionalized magnetic microparticles, fluorescent dye tags, and a custom format flow cytometer, but is not capable of multiplexed assays. Both of these technologies require enzymatic amplification to achieve detection, representing the ability only to observe reaction endpoints, and an assay protocol that requires chemical amplification reagents. The use of NPs (rather than micron-scale beads) for capture and tagging of analytes represents a situation in which a single tag corresponds very closely to a single analyte (because the nanoparticles are nearly the same size as the biomolecules that they tag) for more accurate quantitation. Further, the NP can be simultaneously used as a capture agent and as an imaging contrast agent when combined with a sensing method with single-NP resolution, thus removing the need for enzymatic amplification processes that add time and complexity to assays. In fact, a major advantage shared by all the detection approaches presented in this review is that the sensor generates an output instantaneously with NP capture, which results in immediate output and the capability for dynamically monitoring the accumulation of analytes to yield kinetic information about the processes taking place. This represents a major advantage over methods that require enzymatic amplification, which can only render a single result at the end point of an assay.

In this review, we will restrict ourselves to describing approaches capable of detecting the presence of NPs through their intrinsic dielectric properties, in which the NP’s complex dielectric permittivity, represented by its refractive index and absorption, is responsible for generating some form of contrast. Thus, the modalities we will consider will utilize the interaction of the NP with an optical transducer that enables an externally observable quantity to be detected through, for example, a shift in resonant wavelength, a change in scattered spectrum, or a change in the intensity of reflected/transmitted light due to scattering or absorption. Although nanometer-scale light emitters, such as phosphors, flurophores, and quantum dots [42] also interact strongly with optical nanostructures that can substantially modify their emission properties, they will not be covered in this review and interested readers are directed to several excellent papers on this topic [42–44]. Likewise, this review will not discuss approaches that include surface enhanced Raman scattering (SERS) from molecules attached single nanoparticles, where the reader is referred to [45].

The topics covered in this review are important because single NP detection and the ability to sense many NPs with single-NP (digital) resolution form the underlying principle for novel concepts in biodetection in which a nanometer-sized object (such as a virus particle) is the detection target, or in which a NP is used as a tag to signal when a specific biomolecule-biomolecule interaction has taken place. Direct detection of viruses on an optical transducer that has been prepared with a capture molecule (such as an antibody) that specifically recognizes a protein on the outer coat of the virus is an important capability that is sought for applications that include biological warfare agent defense, HIV viral load monitoring, and food safety. For each of these situations, the number of available pathogens in the test sample may be extremely limited, and thus the ability to capture a large percentage of the pathogens on the active “sensing” region of a transducer is of critical importance, especially in situations in which the presence of even a single virus represents a health threat. Such “direct” detection methods are desirable compared to approaches that require extraction and amplification of pathogen-specific nucleic acid sequences due to potentially improved simplicity (especially through greatly reduced requirement for temperature cycling, extra reagents, and development of “primers” that specifically amplify only the target nucleic acid sequence) and time required to obtain a result. Digital resolution sensing of the presence of specific biomolecules (such as proteins, DNA, or RNA) is an enabling capability for the most demanding applications for in vitro diagnostics such as liquid biopsies for cancer via detection of circulating DNA, messenger RNA (mRNA) or microRNA (miRNA) [46–49], where the test sample volume may be limited to only several microliters (such as a droplet of serum from a fingerstick), combined with analyte concentrations that extend below 1 pg/mL. For such scenarios, the number of available target molecules may be between 100–10,000, and thus approaches that require aggregation of many captured molecules into a compact region, such as a microarray spot, will no longer reliably generate features that can be recognized and quantified [50].

In this review, we will initially categorize sensing approaches by their capability to function as an imaging modality. Non-imaging discrete transducers may be operated either singly or in the form of multiplexed arrays. Generally, the transducers are comprised of a structure that includes a specific region (such as the perimeter of a whispering gallery mode resonator or the cavity of a photonic crystal) in which the electromagnetic field is substantially greater than surrounding regions, representing the location upon which attachment of a nanoparticle will generate the greatest measurable signal. A key challenge for such devices is preparing them so that the target analytes bind only to the sensitive location, rather than becoming bound to a part of the structure where they cannot have an impact on the measured output. Such approaches may have the ability to observe a single nanoparticle that was fortunate to land upon the sensitive part of the transducer, while many thousands of other analytes are present nearby, but not observed. Approaches that are capable of imaging, however, enable counting of each nanoparticle within a field of view, regardless of where they attach to the transducer surface. Thus, the entire transducer surface can be considered as “active” for sensing, and different regions within the field of view can be designated as experimental controls or as regions for multiplexed detection of many analytes.

## 2. Non-Imaging Single Nanoparticle Sensing Methods Materials and Methods

### 2.1. Photonic Crystal Cavities

Photonic crystals (PCs) are periodic dielectric structures that can be designed to generate resonant electromagnetic standing waves at specific wavelengths, at which light cannot propagate freely, as it would in an ordinary nonstructured dielectric material. While the photonic band gap of PCs can forbid light propagation, “defects” such as a missing hole in a periodic lattice of holes, can serve as concentration points for electromagnetic energy.

Recent research efforts have explored the optimization of location, size, and shape of defects in PC structures in order to maximize field confinement for purposes of designing sensors with improved sensitivity for analytes captured near the defect. For further details regarding the electromagnetic principles behind PC defects, readers are referred to [51]. Demonstrated applications include refractive index sensing, optical communication, protein detection, optical trapping, and single nanoparticle detection [52–58].

This section focuses upon single nanoparticle detection using PC cavities, highlighting PC nanobeam cavities (Section 2.1.1), and PC cavities (Section 2.1.2).

#### 2.1.1. PC Nanobeam Cavities

**Figure 1.**
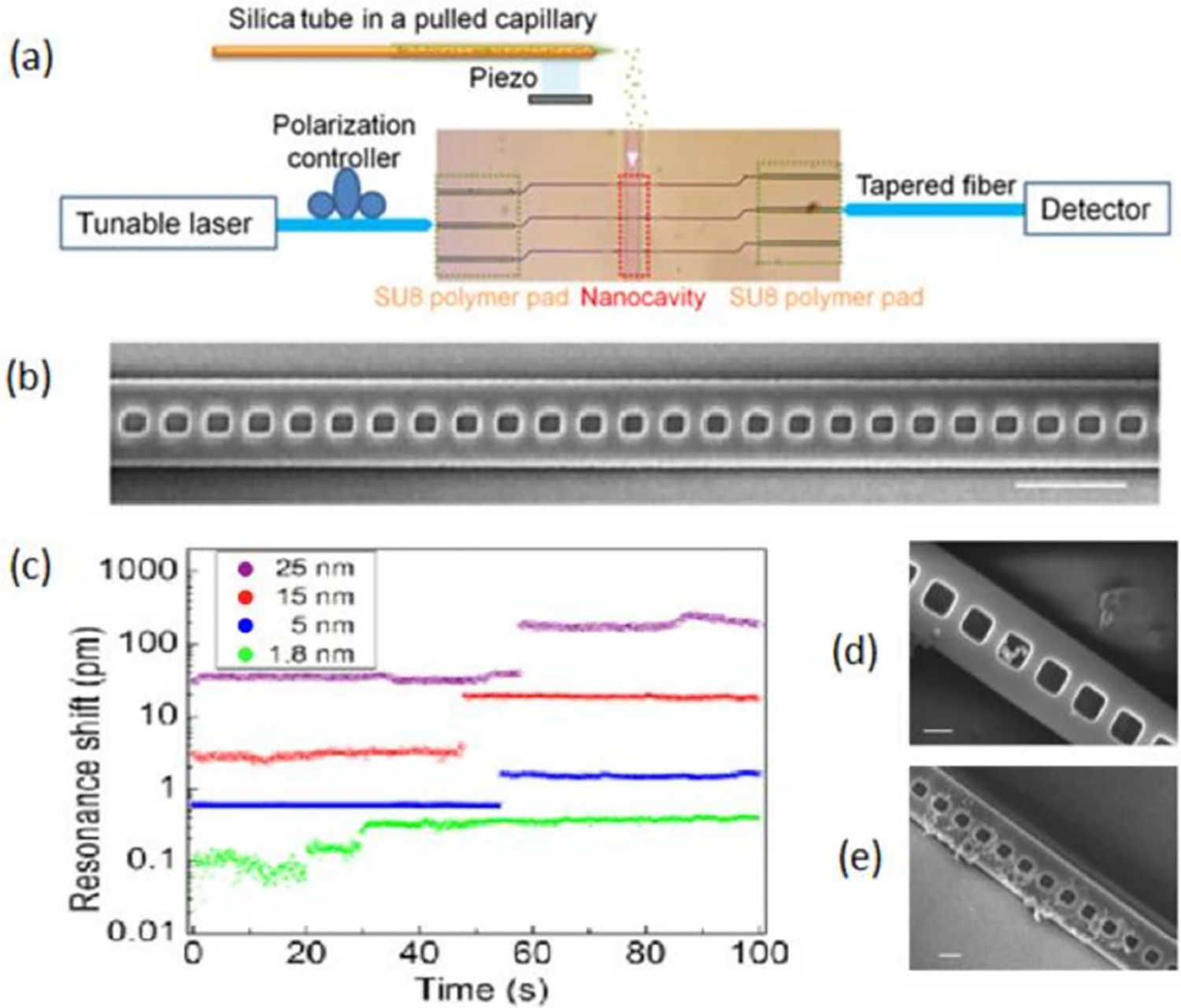
Gold nanoparticle detection system: (**a**) Approach based on the photonic crystal (PC) nanobeam device and piezospray introduction of the test sample; (**b**) Scanning electron microscopy (SEM) image of the nanobeam cavity (top view). Scale bar: 1 μm; (**c**) resonance shifts caused by attached nanoparticles with diameters of 25 nm (purple), 15 nm (red), 5 nm (blue), and 1.8 nm (green); (**d**) SEM image of nanoparticles deposited in the center region when the cavity resonance was excited; (**e**) SEM image of nanoparticles deposited randomly along the waveguide when the laser is off. Scale bar: 200 nm. Adapted with permission from reference [59]. Copyright (2015) American Chemical Society.

A sensor and detection instrument based upon a photonic crystal nanobeam cavity was proposed by Liang and Quan for detection and sizing gold nanoparticles down to 1.8 nm diameter [59]. As shown in Figure 1a, the system is comprised of: a piezoelectric device for nebulizing a nanoparticle solution in methanol, a tunable laser scanning its wavelength from 1420 nm to 1520 nm, a polarization controller, two tapered optical fibers, one coupled the light from the laser to a patterned polymer pad (made of SU8) and the other coupled the light from the pad to the detector; and finally, the PC nanobeam cavity. A scanning electron microscopy (SEM) image of the nanobeam cavity is shown in Figure 1b.

The nanobeam cavity consists of an array of rectangular gratings along an 800 nm wide silicon waveguide. The distance between two adjacent gratings is fixed at 400 nm; the width of each rectangular grating is fixed at 200 nm. The lengths of the rectangles are linearly increased from 220 nm to 240 nm from the middle of the nanobeam cavity to each of its ends. The tapering geometry is optimized to create a hyperbolic potential for photons of a specific wavelength, thus confining their electromagnetic energy to the middle of the structure with a Gaussian distribution [60].

In this system (Figure 1a), the sensor interfaces with the external detection instrument through tapered optical fibers to illuminate the structure via a tunable infrared laser at one end and a detector measuring transmitted light on the opposite end. The system’s gradient in PC period generates strong optical field gradients, which draw nanoparticles to the center of the cavity and once inside, push them towards the side wall (Figure 1d). When the nanoparticles are adsorbed in the sensor’s active region, the resulting change in the refractive index of the material surrounding the resonator is measured by recording a shift of the resonant wavelength of the PC. On the other hand, when the laser is turned off, nanoparticles deposit randomly along the waveguide (Figure 1e). The authors reported discrete resonance wavelength jumps when gold nanoparticles (i.e., 25, 15, 5, and 1.8 nm of diameter) were deposited into the cavity (Figure 1c) [59]. The authors claim that the low detection limit (i.e., 1.8 nm diameter) is achieved because the improvement of the Q-factor (i.e., 2.5 × 10^5^) and the cavity mode volume (0.01λ^3^) at high frequencies (200 THz).

Resonance shifts were also observed when Ag nanoparticles with a diameter of 80 nm were trapped by a PC nanobeam cavity [37]. In a separate study, Mandal et al. [61] demonstrated the ability to transport, trap and manipulate nanoparticles using a photonic crystal nanobeam cavity. These authors reported, for the first time, the use of a one dimensional PC cavity in the form of a nanobeam where they could trap particles with diameters as small as 48 nm. Liang et al. [62] demonstrated label-free nanoparticle detection and protein detection with single particle sensitivity. Using this approach, the authors confirmed the detection of polystyrene nanoparticles with diameters as small as 25 nm.

Additional PC nanobeam cavity devices based upon similar principles have been recently reported: Renaut et al. [63] demonstrated a nanobeam cavity consisting of a waveguide region for particle trapping, and both Wang et al. [64] and Lin et al. [55] designed a slotted PC nanobeam cavity for nanoparticle detection. As a result, several PC cavity designs [65,66] have been investigated for particle confinement as they constrain light in narrow waveguide geometries, thus creating ultrahigh electromagnetic (EM) field gradients for nanoparticle trapping and detecting.

#### 2.1.2. PC Cavities

An enormous variety of sensor designs using PC cavities have been demonstrated through engineering the dimensions, number, and arrangement of defects, or the dimensions of the PC. The remaining designs that will be discussed in this section rely on one or more of these alterations to the PC period in order to produce a strong EM-field confinement in a localized region. These approaches may be categorized along three design principles: (i) point-like defect, (ii) waveguide defect, and (iii) slotted waveguide defect.

As an exception, Baker et al. [67] utilized a device that introduced local random disorder in the refractive index distribution (called a defect-free PC cavity) to demonstrate size-selective detection of latex particles. In their experiments, the infiltration of latex particles with diameters of 260 nm and 320 nm within the PC lattice holes of 280 nm in diameter was investigated. A significant red-shift in the band-edge of the crystal was observed for the infiltration of smaller particles, thus demonstrating size selective particle detection. With this PC structure, a detection limit of <200 particles was achieved and a theoretical limit of detection of <10 particles was reported.

##### Point-Like Defects

**Figure 2.**
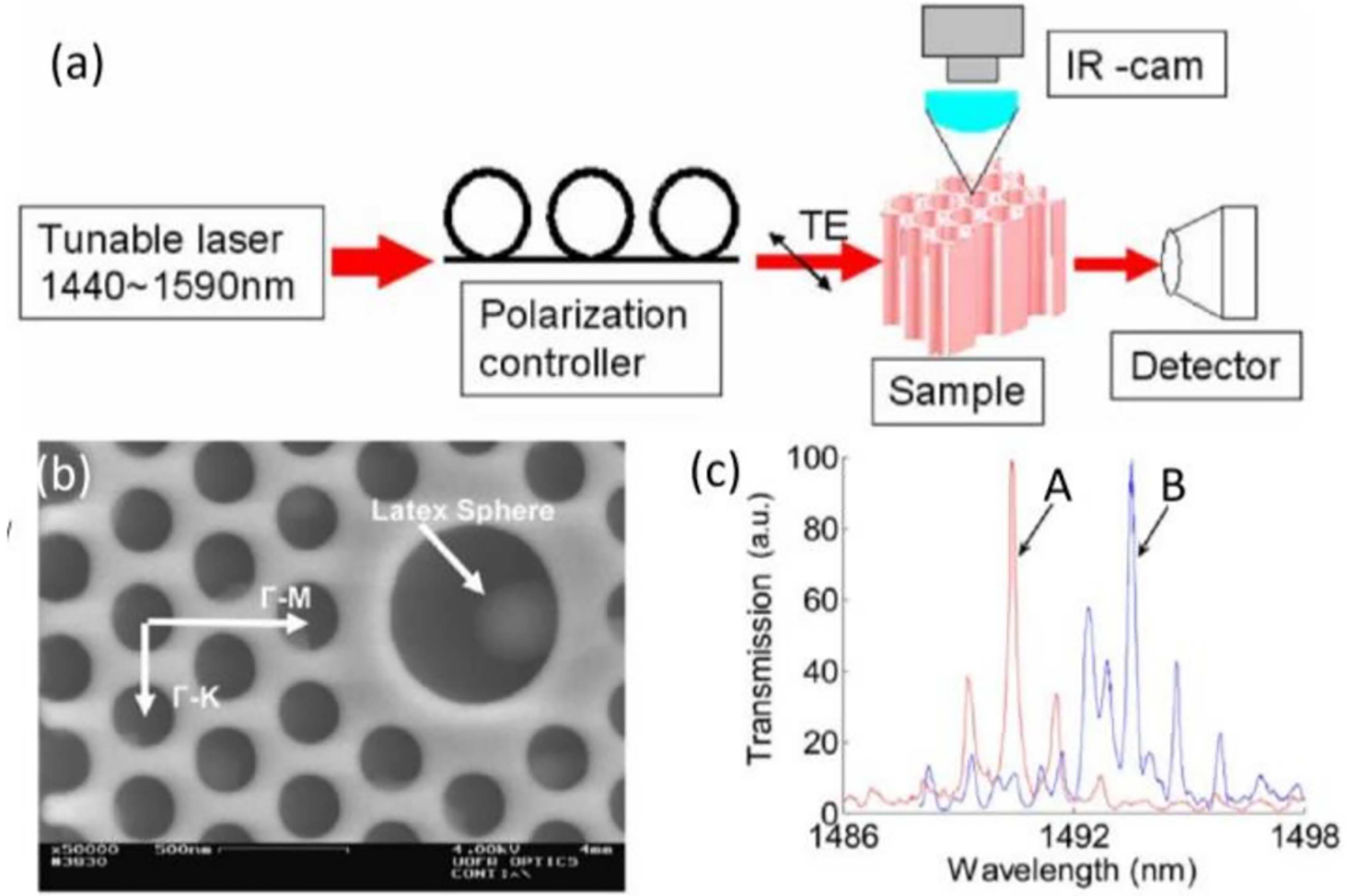
Schematic of the experimental setup: (**a**) A tunable laser (1440 to 1590 nm) is used as the source, and TE-polarized light is generated using a polarization controller plate. Light is coupled in and out of the PC through ridge waveguides. An InGaAs detector is used to measure the transmitted signal; (**b**) top view of the device with one latex sphere captured in the central defect; (**c**) normalized transmission spectra; Curve A is measured before capture, and curve B is measured after one latex sphere is infiltrated inside the defect. Adapted with permission from reference [70]. Copyright (2007) Optical Society of America Publishing.

Changing the size [68–72], location [73], shape [74], or presence of [75] one or more lattice points, or even the combination of these [76,77] can create a strong electric field localization around that altered region. By making these modifications within a small region of the PC, several research groups used point-like defects to detect nanoparticles. For example, a single latex sphere was detected by Lee and Fauchet using the experimental setup shown in Figure 2a [70]. In this research, a tunable infrared laser was used as an illumination source and the transverse electric (TE) polarized beam was focused onto the waveguide via a tapered, lensed, polarization-maintaining fiber. Finally, the transmitted signal was measured using an InGaAs detector. In addition, an infrared camera was used for alignment and monitoring the radiation losses.

The structure of this PC is illustrated in Figure 2b. The PC consists of a hexagonal array with a lattice constant *a* = 400 nm and a pore diameter *d* = 240 nm. The defect is introduced by increasing the center pore diameter to *d* = 685 nm (i.e., approximately three times larger than the surrounding holes). The authors captured one latex sphere (*d* = 370 nm) inside the microcavity and measured the transmission spectrum before and after capture (Figure 2c), showing a redshift in the resonance of approximately 4 nm. The authors concluded that the device can be used for detecting single particles that have a diameter of ≤50 nm [70].

Recently, a new technology has been developed combining the performance of PC cavity biosensors with the unique advantages of optical fibers. The ability of optical fibers to conduct light from a sensing region to a remote location, makes them an ideal platform to perform measurements in highly confined spaces. “Lab on a Fiber” [54] technology is aimed at developing innovative types of optical fiber probes with extraordinary features in terms of miniaturization levels and functionalities, especially ones exploitable for biological sensing. In particular, we wish to highlight recent work in which a PC is produced upon the tip of an optical fiber. In this arrangement, excitation light and reflected light can be introduced and detected from the distal end of the fiber, while the active sensor region on the proximal end of the fiber may be inserted into the sample media. Shambat et al. [78,79] demonstrated a simple epoxy-based technique for integrating PC cavities onto the tips of optical fibers in which semiconductor PC cavities were transferred to the fiber tip. This fiber-PC device is has been used as a sensor for both iron oxide and gold nanoparticles [78].

##### Waveguide Defect

One of the most common methods of producing PC defects is the removal of a single row of holes from the crystal, thus creating a line defect structure or waveguide defect [80,81].

This waveguide defect structure has been studied by several research groups for sensing applications. Notomi et al. demonstrated that it is possible to realize a variety of coupled elements within PC waveguides [82]. Other types of PC waveguide cavities have also been reported, including a PC waveguide with line defects connected as a hexagon [83] and a double-heterostructure cavity in which the lattice constant was changed [76,84].

**Figure 3.**
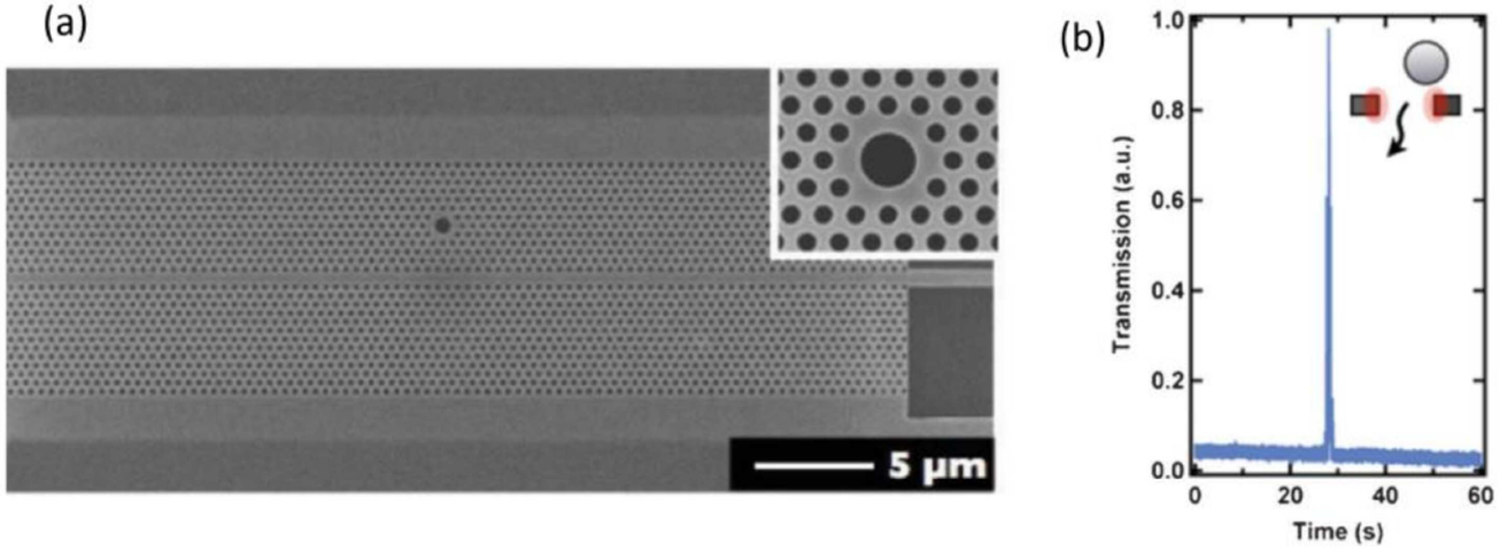
(**a**) Scanning electron micrograph of a PC structure, lattice constant 420 nm, showing the large point defect side-coupled with the PC waveguide defect; (**b**) experimental record of transmission through the structure when a particle is moving through the cavity as illustrated in the inset. Adapted with permission from reference [86]. Copyright (2013) Royal Society of Chemistry.

By introducing defects into the lattice, high quality factor cavities can be created, allowing smaller wavelength shifts (induced by captured nanoparticles) to be resolved more easily. For instance, Descharmes et al. [85,86] reported single particle detection using a PC waveguide cavity with a single large point defect in a hexagonal lattice (Figure 3a). In their experimental setup, it is possible to record large dynamic changes in the power transmitted through the device, while the particle traverses the PC waveguide cavity, as can be observed in Figure 3b. This approach demonstrated detection and manipulation of sub-μm diameter particles (i.e., polystyrene particles with the diameters of 250 and 500 nm). They also postulated that the simultaneous trapping and induced resonance shifts have potential applications for virus detection.

With a similar PC cavity structure, Baker et al. [87] confirmed the ability of their device to detect virus-sized particles under flow via a recognition-mediated process. In this approach, a PC waveguide cavity with a large point-like defect was functionalized with IgG antibodies and integrated with a simple microfluidic channel which produced an optical response when exposed to virus-sized, anti-IgG-functionalized particles under flow. They employed antibody-functionalized latex particles with a pre-conjugation diameter of 320 nm (similar to the dimensions of vaccinia or variola viruses).

A further example of this strategy was the utilization of a reduced point-like defect coupled to waveguide defect. Using this approach, Pal et al. [88] demonstrated sensing of virus-like nanoparticles.

##### Slotted Waveguide Defect

Another important category of PC defect waveguides is the slotted waveguide defect, in which a thin line of dielectric material is removed along the center of the waveguide over its entire length. Almeida et al. [89] demonstrated that a line defect structure inside a waveguide can be used to strongly enhance and confine light. This concept was implemented in a number of successful experimental demonstrations of slab PC cavity designs [90–93] where several approaches were used to promote strong light-matter interactions. For nanoparticle applications, the slot waveguide PC cavity was used to stably trap nanoparticles with low input power. Several near-field trapping configurations can increase the field amplitude inside the device and create a large spatial EM-field gradient [94]. For instance, Lin and Lee proposed a slot waveguide PC cavity design for utilizing slow light [95].

### 2.2. Whispering Gallery Mode Sensors

Whispering gallery mode (WSG) sensors are a family of microresonators in which light-matter interactions are significantly enhanced by confining light within an extremely-limited volume. The term “whispering gallery mode” was originally used to describe the acoustic resonance phenomenon in the gallery of St. Paul’s Cathedral, and the concept was further extended to the field of optical resonators. A typical WSG sensor has a circular cross-section as viewed from above, and total internal reflection occurs as the light travels around the perimeter. The structure of the WSG sensor allows light at specific modal wavelengths (defined by integer numbers of half wavelengths that fit around the perimeter) to travel repeatedly around the perimeter without suffering significant losses, resulting in a high quality factor (or *Q* factor, where *Q* = *ω*_0_/*γ*_0_, *ω*_0_ is the resonant frequency of the cavity and *γ*_0_ is the full-width at half-maximum of the spectrum). It has been reported that a *Q* factor as high as 10^10^ can be achieved for wavelengths within the visible spectrum [96]. With these high *Q* factors, even slight changes to the WSG sensor’s effective refractive index (such as those introduced by a temperature change or the attachment of a nanoparticle) will affect the modal frequency of the traveling wave, which can be measured by a shift in the wavelength of light optimally coupled from an adjacent waveguide to the WSG ring. The presence of a nanoparticle on the perimeter of the WSG sensor is also capable of scattering light which can propagate in the reverse direction, causing a single initial WSG mode to split into multiple counterpropagating modes. The ability to resolve modal wavelength shifts ranging from 0.1 pm to several nanometers, in combination with extremely small modal volumes, has generated broad interest in WSG-based sensing, with a variety of geometries such as spheres [97–99], droplets [100], disks [101–106], rings [107,108], bottles [109], bubbles [110–112], and capillaries [113,114].

#### 2.2.1. WSG Sensing Principle

**Figure 4.**
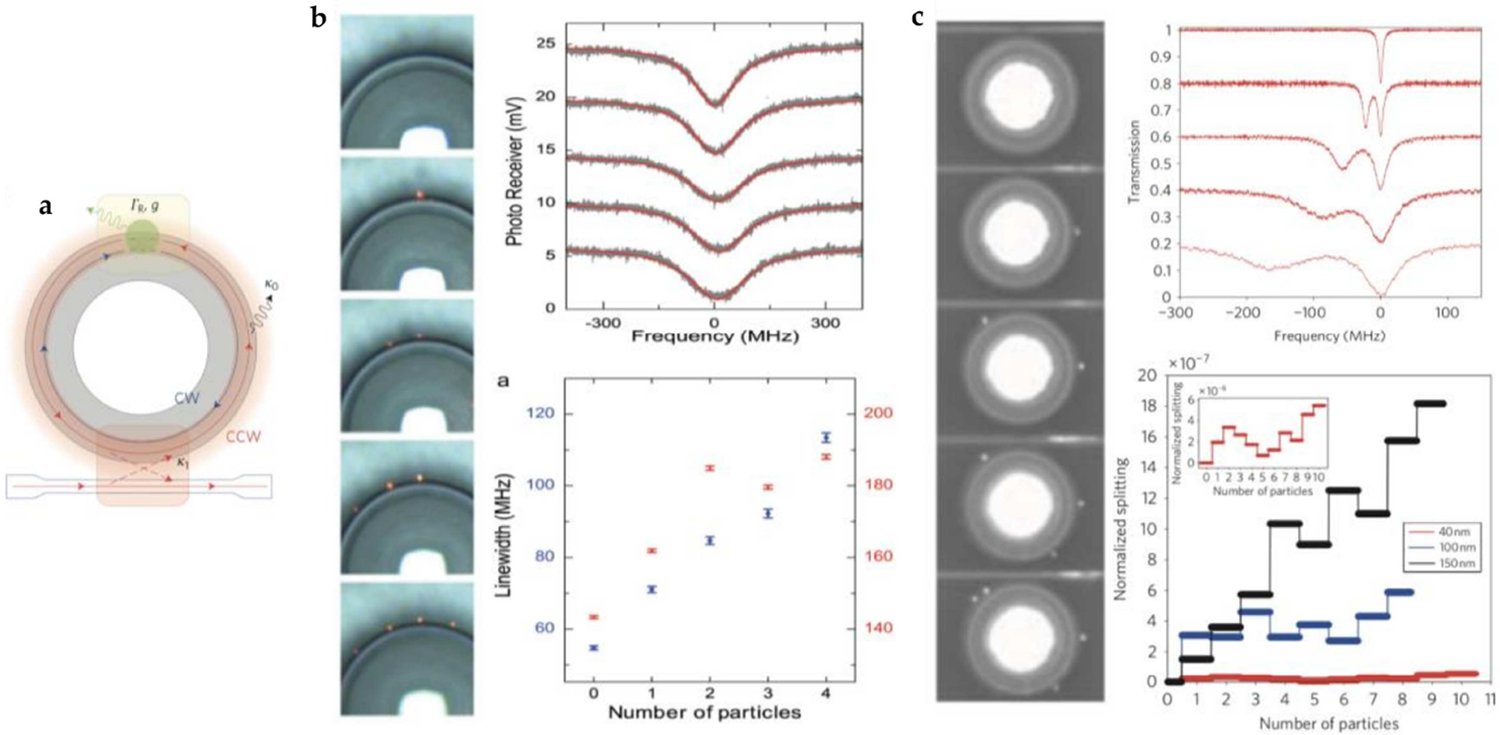
(**a**) Schematic of the microtoroid resonator in the presence of a nanoparticle. A portion *g* of the light scattered by the nanoparticle is coupled back into the resonator, allowing both CW and CCW propagating light in the cavity. Reproduced with permission from [21]. Copyright (2010) Nature Publishing Group. (**b**) Transmission spectra and the corresponding optical images attachment of four successive individual nanoparticles. The normalized spectra are vertically shifted for clarity. Normalized mode splitting versus single nanoparticle binding events are shown, with the inset representing the enlarged plot for nanoparticles at *R* = 40 nm. Different sizes of nanoparticles are also examined, and represented by different colors in the figure. Reproduced with permission from [21]. Copyright (2010). Nature Publishing Group. (**c**) Mode broadening induced by four successive polystyrene nanoparticles at *R* = 70 nm. Linewidths of the two different WSG sensors in the experiment are shown in the lower diagram. Reproduced with permission from [120]. Copyright (2013) WILEY-VCH Verlag GmbH & Co. KGaA, Weinheim.

Frist demonstrated by Arnold et al. in 2002, WSG sensors were considered for detecting the presence of specific molecules, such as protein, with exceptionally high sensitivity [97,115]. Depending on the properties of the detected analyte, WSG sensors demonstrate several potential spectral responses including frequency shift, line broadening, mode splitting, and fluctuation of reflected light intensity. As indicated in Figure 4a, due to the geometric symmetry of microtoroid WSG sensors, both clockwise (CW) and counter-clockwise (CCW) propagating waves can be supported in the cavity, but only one is sustained, depending on the incident direction of the coupled incident light [103,116]. However, in the presence of an attached scattering molecule, backscattered light can again be coupled in the WSG sensor and thus both CW and CCW propagating waves can coexist and interfere with each other, resulting in resonant frequency shifting, mode splitting or broadening. Furthermore, if the scattering molecule is also absorptive at the resonant wavelength, a portion of photon energy is lost due to absorption, and correspondingly the line shape of the WSG sensor will be broadened.

##### WSG Sensor Frequency Shift

Detection of resonant wavelength shifts is the mechanism most conventionally utilized for WSG sensors, which occurs when the refractive index of the surrounding medium changes. By the virtue of first order perturbation theory, the fractional angular frequency shift induced by a particle much smaller than the probe wavelength is given by [115,117]:

$$\frac{\delta \omega }{\omega }≅-\frac{\alpha {\left|E_{0}\left({\vec{r}}_{p}\right)\right|}^{2}}{2\int \epsilon_{0}\epsilon_{r}(\vec{r}){\left|E_{0}(\vec{r})\right|}^{2}dV}$$

where *α* is the polarizability of the particle, $E_{0}\left({\vec{r}}_{p}\right)$ and $E_{0}(\vec{r})$ are the magnitude of the electric field at the location of the attached particle and of the overall resonant cavity, $\epsilon_{0}\epsilon_{r}(\vec{r})$ is the permittivity of the material of the overall cavity, respectively. It has been shown that the frequency shift is not only dependent on properties of the particle but also on the relative position between the particle and the WSG sensor.

In 2008, the first WSG-based single nanoscale particle detection experiment was reported by Keng et al. [117]. A microsphere is used to support WSG modes and to detect the absorption of single influenza A (InfA) virus particles with a diameter of 50 nm. Discrete changes induced by single viruses in the resonant wavelength shift can be clearly observed. By incorporating a nanoplasmonic enhancing effect into a WSG sensor, Arnold et al. further pushed the sizing and detection to the limit, by measuring attachment events of the smallest RNA virus, the MS2 bacteriophage (5 nm diameter) in a microcavity with modest Q factor [118]. The resultant frequency shift is amplified by 70 times by virtue of the nanoshell plasmonic dipole; however, detection based on frequency shift usually suffers from laser frequency noise and thermal fluctuations, thus it requires rigorous environment control such as high vacuum and temperature control [119].

##### WSG Mode Splitting

Considering the limits of direct frequency shift, self-referenced mode splitting as a merging sensing mechanism has attracted a significant amount of attention in the field of WSG sensors, mainly due to its comparative insensitivity to environmental factors. As mentioned previously, a symmetric WSG sensor can potentially support modes in the counter-propagating direction. As scattering particles are introduced to the light path, a doublet will appear in the spectrum. A portion of light is scattered and travels in the counter-propagating direction, lifting the degeneracy of CCW waves. Such a phenomenon has been described using an analytical model [21,116,121], in which the coupling strength δ and the additional damping Γ_R_ of the splitting are respectively given by:

$$\delta =-\frac{\alpha f^{2}(r)\omega_{0}}{V}$$

$$\Gamma_{R}=\frac{\alpha^{2}f^{2}(r)\omega_{c}^{4}}{6\pi c^{3}V_{c}}$$

where *α* is the polarizability of the scattering nanoparticle, *f*(***r***) is the overlap of the light field between the WSG sensor and the nanoparticle, *ω*_0_ is the resonant frequency of the sensor, *ω*_*c*_ denotes the degenerate angular frequency between CW and CCW waves, and c is the speed of light while *V*_*c*_ is the cavity volume. Although both coupling strength and additional damping are both dependent on the relative location of the particles with respect to the WSG sensor, such location dependence can be removed by dividing the two terms [21]:

$$\frac{\delta }{\Gamma_{R}}=\frac{8\pi^{2}\alpha }{3\lambda^{2}}$$

which is merely dependent on the polarizability of the particles and immune to thermal fluctuations and pressure changes.

The mode splitting effect induced by defect particles in WSG mode structures was observed as early as 1995 [122]. In 2009, Yang et al. demonstrated the ability to estimate the sizes and the number of nanoparticles attached to a WSG microtoroid sensor, by measuring the splitting of modes in air [21]. In Figure 4b, a series of normalized transmission spectra are shown corresponding to the optical images. It is observed that with the addition of nanoparticles, the original Lorentzian resonance is deformed and a doublet mode form, enabling the differentiation of individual nanoparticles of different sizes.

##### WSG Sensing by Line Broadening

Perturbations to the WSG sensors will also induce line broadening effect through several possible physical mechanisms that include both modulation of the coupling strength and losses due to scattering. Figure 4c demonstrates the effect of nanoscale scattering particles on the transmission spectrum of WSG sensors [120]. Individual polystyrene nanoparticles of 70 nm diameter were added to a torodial cavity with Q factor of ~10^6^, demonstrating that as the nanoparticles are adsorbed on the toroid, the corresponding line shapes gradually become broader. Detection by measuring line broadening retains the advantages of sensing via mode splitting, yet measuring changes in the resonant linewidth does not require a sensor with such a high Q-factor [123].

## 3. Imaging Methods

### 3.1. Surface Plasmon Resonance Imaging Microscopy

Surface plasmonic resonance (SPR) imaging microscopy is a refractive index-based imaging technique with the capability to detect and characterize the properties of single nanoparticles. For SPR imaging, the sensing transducer is comprised of a glass slide that is uniformly coated with a thin layer of plasmonic metal (i.e., Au, Ag and Cu, etc.) which, when excited by a light source that matches the wavelength, angle, and polarization of the SPR coupling condition, generates traveling surface plasmon polariton (SPP) waves on the metal surface. A nanoparticle in close proximity to the metal surface will act as a highly localized scatterer, and therefore create a point-diffraction pattern on the surface plasmon wave, which can be directly captured and recorded using a charge-coupled device (CCD) camera.

**Figure 5.**
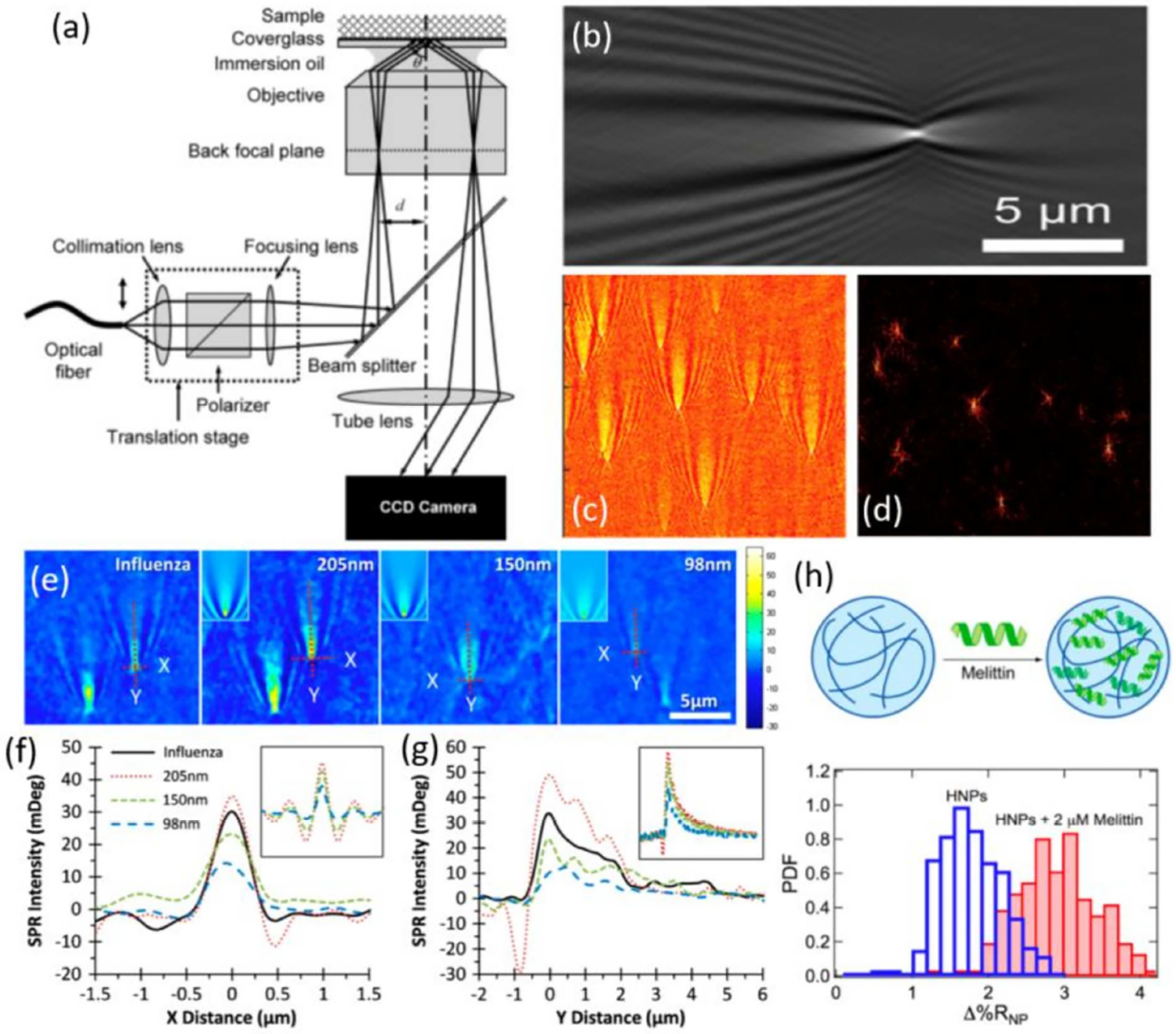
(**a**) Configuration of a surface plasmonic resonance (SPR) imaging microscope. Reproduced with permission from [128]. Copyright (2007). American Chemical Society. (**b**) SPR image of a polystyrene nanoparticle at R = 85 nm. Reproduced with permission from [133]. Copyright (2017). American Chemical Society. (**c**) SPR images of polystyrene nanoparticles at R = 50 nm. (**d**) Corresponding reconstructed SPR images with improved lateral resolution. (**c**,**d**) are reproduced with permission from [139]. Copyright (2017). American Chemical Society. (**e**) SPR images of InfA and silica nanoparticles of different sizes. (**f**) and (**g**) Intensity profiles of the SPR patterns along X and Y directions, respectively. (**e**–**g**) are reproduced with permission from [24]. Copyright (2010) National Academy of Sciences. (**h**) Frequency distribution of average single nanoparticle responses of Hydrogel nanoparticles at the absence and presence of 2 μM of melittin. Reproduced with permission from [133]. Copyright (2017). American Chemical Society.

The Kretschmann configuration for SPR imaging utilizes a prism for light coupling [124], allowing incident light from a laser or light-emitting diode (LED) to launch SPP waves onto the sensing surface [125]. However, prism-based systems can be physically bulky and limited in their spatial resolution, and their long working distance can produce image distortion [126,127]. To address these issues, Zare et al. first demonstrated SPR imaging using a high numerical aperture objective, as shown in Figure 5a [128]. Collimated and polarized light was focused on the back focal plane of the objective, which illuminated the gold-plated glass substrate at a specific angle as a collimated beam. Reflected light from the sample was recaptured by the objective and projected onto a CCD camera, resulting in imaging resolution close to the diffraction limit.

Using this higher resolution, Tao et al. first reported the label free detection, imaging and mass measurements of single viruses using an SPR imaging microscope in 2010 [24]. A variety of nanoscale particles, such as metallic nanoparticles [129], dielectric nanoparticles [130,131], protein nanoparticles [132,133] and single DNA molecules [134,135] have been observed with this system. In addition, orthogonal and complementary measurement techniques, such as electrochemistry [129,136,137] and local thermal measurement [138], have also be incorporated into the system.

#### 3.1.1. Imaging Principle

Numerous publications describe the underlying theory behind SPR imaging [140,141]. Here, we present a comprehensive, but simplified model. First, a metallic surface is illuminated by incident light at a specific angle, thereby generating a plasmonic wave propagating along the surface. A nanoparticle placed in close proximity to the metallic surface will scatter the propagating surface plasmonic wave. The total reflected light intensity can be captured by a CCD camera and can be represented by the superposition of the reflected surface plasmonic wave *E*_*p*_ and the scattered field *E*_*sc*_:

$$I\propto {\left|E_{p}+\beta E_{sc}\right|}^{2}$$

where *I* is the intensity of SPR image, and *β* is the portion of the scattered plasmonic wave captured by the camera. The reflected surface plasmonic wave *E*_*p*_ can be accurately calculated using Fresnel’s equation based on stratified medium model [142,143]. Since the size of the nanoparticle is much smaller than the surface plasmon wavelength, the scattered field *E*_*sc*_ can be described using the elastic scattering theory:

$$E_{sC}=\alpha E_{p0}e^{-\gamma r}e^{-jk_{0}r}$$

where *α* is the polarizability of the nanoparticle, *γ* is the attenuation coefficient of the surface plasmon wave, *k*_0_ is the wavevector of the plasmon wave, and *r* is the distance to the scatterer. It can be observed from the above expressions that the interference between the scattering field and the propagating plasmonic wave together creates a distinct parabolic fringe pattern. Figure 5b is an SPR image of a 170 nm diameter polystyrene nanoparticle absorbed to a functionalized gold surface, which demonstrates this characteristic parabolic pattern. It is worth noting that the parabolic tail extends 10 μm in the longitudinal direction, which is large relative to the size of the scatterer. Thus, the lateral resolution of SPR imaging is greatly limited by decay distance of parabolic tails of the plasmon wave in the propagation direction, especially in the case where other nanoparticles may be within the propagation length of the surface wave.

#### 3.1.2. Spatial Resolution and Image Reconstruction

To overcome the spatial resolution limitations of SPR imaging for individual nanoparticle detection, efforts have been reported to either decrease the decay length, or to mathematically reconstruct SPR images through complex field deconvolution.

To shorten the decay length, one of the simplest solutions is to increase the damping of the SPR wave either by using a lossy metal surface or by using light of a shorter wavelength. According to effective medium theory (EMT) [142], introducing nanostructures onto the surface, such as periodic subwavelength gratings [144], can also improve the lateral resolution as it prevents localized surface plasmons from coupling into propagating SPR waves. On the other hand, Zare et al. has shown that when the wavelength of excitation light switches from 638 nm to 532 nm, the propagation length on gold-plated surface can be reduced from 8.3 μm to 0.5 μm (in air), without significantly compromising the refractive index sensitivity [128]. Finally, by focusing the laser beam to a narrow line and scanning the laser line across the surface, the lateral resolution can be greatly improved, though it decreases temporal resolution for time-course scanning due to the scanning process [145,146].

Another important technique to restore lateral resolution is via image reconstruction. As mentioned previously, the characteristic parabolic patterns of nanoparticles in SPR imaging results from the interference between the travelling plasmonic wave on the metallic surface and the scattered EM-field of nanoparticles. Inasmuch as the scatterer is in the single scattering regime [125] (i.e., the scattered field can be described as the convolution of point spread function and the spatial distribution of refractive index of the nanoparticle), high resolution images of the nanoparticle can be obtained by 2D Fourier transform and complex field deconvolution. As recently reported [139], near-diffraction-limit-resolution for both longitudinal and transverse direction can be achieved by applying such a reconstruction method. In Figure 5c,d, original SPR images of 100 nm polystyrene nanoparticles and the corresponding reconstructed images are respectively shown, in which the parabolic tails are removed and each nanoparticle is represented by a bright spot. The achieved spatial resolution is estimated to be ~310 nm, which is close to the diffraction limit of the optical system (230 nm). Importantly, this image reconstruction technique does not require any modification to the optical setup or the acquisition of multiple images, thus it offers a convenient solution to improve lateral resolution while maintaining high temporal resolution. In addition, such a reconstruction method is also capable of resolving strongly interfered diffraction patterns when particles are in close proximity.

#### 3.1.3. Nanoparticle Sizing and Specification

Similar to other SPR-based sensing techniques, the intensity of a single diffraction pattern in SPR imaging depends on the overall refractive index of the corresponding nanoparticle, and is therefore related to both nanoparticle size and material [147–150]. In Figure 5e, silica nanospheres of different sizes (98 nm, 150 nm and 205 nm) and influenza viruses were observed with an SPR imaging microscope, with the corresponding intensity profiles parallel to and perpendicular to the surface plasmon propagation direction shown in Figure 5f,g [24]. It is worth noticing that, although the size-dependent optical responses of these dielectric nanoparticles and viruses are not as large as those obtained from metallic or semiconductor nanoparticles [150,151], significant intensity variation among these nanoparticles can still be observed, especially near the center of the diffraction pattern.

Compared with other non-imaging SPR techniques, SPR microscopy has the advantage that it can resolve each individual nanoparticle, and thus facilitate multiplexed nanoparticle analysis. SPR imaging microscopy is especially powerful in the field of polymeric and protein nanoparticle (PPNP) detection, where high throughput and high sensitivity real-time characterization of dielectric nanoparticles is required. Corn et al. recently utilized SPR imaging microscopy to characterize the size, material content and the inter-particle interactions of PPNPs, in which changes in the intensity of average single-nanoparticle surface plasmon resonance image (SPRI) response (Δ%R_NP_) at the center of the diffraction pattern is used to quantify the bioaffinity uptake of polypeptides and proteins by a variety of solid, porous PPNPs [133]. Figure 5h shows the frequency distribution histogram of the SPR responses of NIPAm-based hydrogel nanoparticles (*d* = 272 nm) in both the absence and presence of melittin. With the uptake of melittin, the hydrogel nanoparticles demonstrate an overall increase in absorption. By measuring the SPR responses of individual nanoparticles, SPR microscopy provides an overview of the extent of bioaffinity-based uptake, which would otherwise be unmeasurable with microscopy.

### 3.2. Interferometric Reflectance Imaging Sensor

Interferometric Reflectance Imaging Sensor (IRIS) is a spectroscopic imaging biosensor approach which measures the interference of fields reflected from a surface comprised of a silicon dioxide layer on top of a silicon surface. IRIS has proven to be a high-throughput imaging technique for measuring binding kinetics on a sensor surface [152], and has been adapted to array formats for multiplexed assays. There are two distinct modalities for IRIS: low magnification to measures biomass accumulation and high magnification to digitally detect single nanoparticles and viruses.

#### 3.2.1. Low Magnification IRIS

The low magnification IRIS system measures biomass accumulation on the SiO_2_/Si surface, based on the spectral reflectivity. The accumulation of biomass on the SiO_2_/Si surface increases the thickness of the upper layer thereby causing an increase in the optical path difference (OPD) between the substrate’s top surface and the SiO_2_/Si interface [153]. The increased OPD induces a quantifiable shift in spectral reflectivity and the sensitivity of this system is 4 pg/mm^2^ [154]. A CCD samples reflectivity curves at different wavelengths and spectral reflectance signatures are fitted using Fresnel equations [153]. With a single image, the system can capture a region of interest containing thousands of biomarkers in 30 s using an external tunable laser to illuminate the SiO_2_/Si surface. Nelsonian illumination, augmented with rotating glass disks to remove speckles from the laser, is used to provide uniform illumination intensity. Any fluctuations in the incident intensity are monitored by a photodetector. Prior to each experiment, a baseline intensity is measured and spatial fluctuations are corrected. The photodetector will measure any fluctuations during data acquisitions and its measurement and the mirror image will be used to normalize the data. Applications of the low magnification IRIS system include: DNA quantification and protein adsorption, measurements of association and disassociation rates and real-time observation of binding kinetics.

#### 3.2.2. High Magnification IRIS

The high magnification IRIS system or single particle IRIS (SP-IRIS) was adapted from the low magnification IRIS system. For the detection and sizing of viral nanoparticles using SP-IRIS, an LED illuminates the SiO_2_/Si sensor surface containing immunocaptured viruses or nanoparticles of interest. The presence of particles on the sensor surface modifies the interference pattern of light reflected from the sensor resulting in a distinct signal that is captured by the camera [154]. A captured nanoparticle is shown as a dot in the image and the size of the particle can be calculated based on the image contrast. The ability of this system to perform particle sizing enables discrimination of impurities such as aggregates and dust particles. Recently, the SP-IRIS system was modified to incorporate polarizing optics to provide enhanced contrast for the detection of metallic particles such as gold nanorods or nanospheres [153]. The use of functionalized nanoparticles can increase the sensitivity of this system and enable single molecule or particle quantification [153].

#### 3.2.3. Evolution of the IRIS System

The first implementation of the IRIS system (low magnification) used a very expensive external cavity tunable laser source (~$20,000), an external photodetector, and the rotating glass discs as previously described. Furthermore, the limited spectral range requires the SiO_2_ layer to be 5 μm thick, thereby increasing the manufacturing costs of the wafers; also, the electronics of this system were bulky. The IRIS system has undergone several improvements since its first design, such as the switching of the laser source to a multi-wavelength LED source, which also eliminated the need for the rotating glass discs used to reduce laser speckle. The SiO_2_ thickness of the IRIS substrate was reduced from 5 μm to 500 nm resulting in a further decrease in the cost, size and complexity of the system [152]. Such changes to the optical path of the IRIS system has enabled single molecule detection capabilities in the high magnification SP-IRIS.

### 3.3. Interferometric Scattering Microscopy

**Figure 6.**
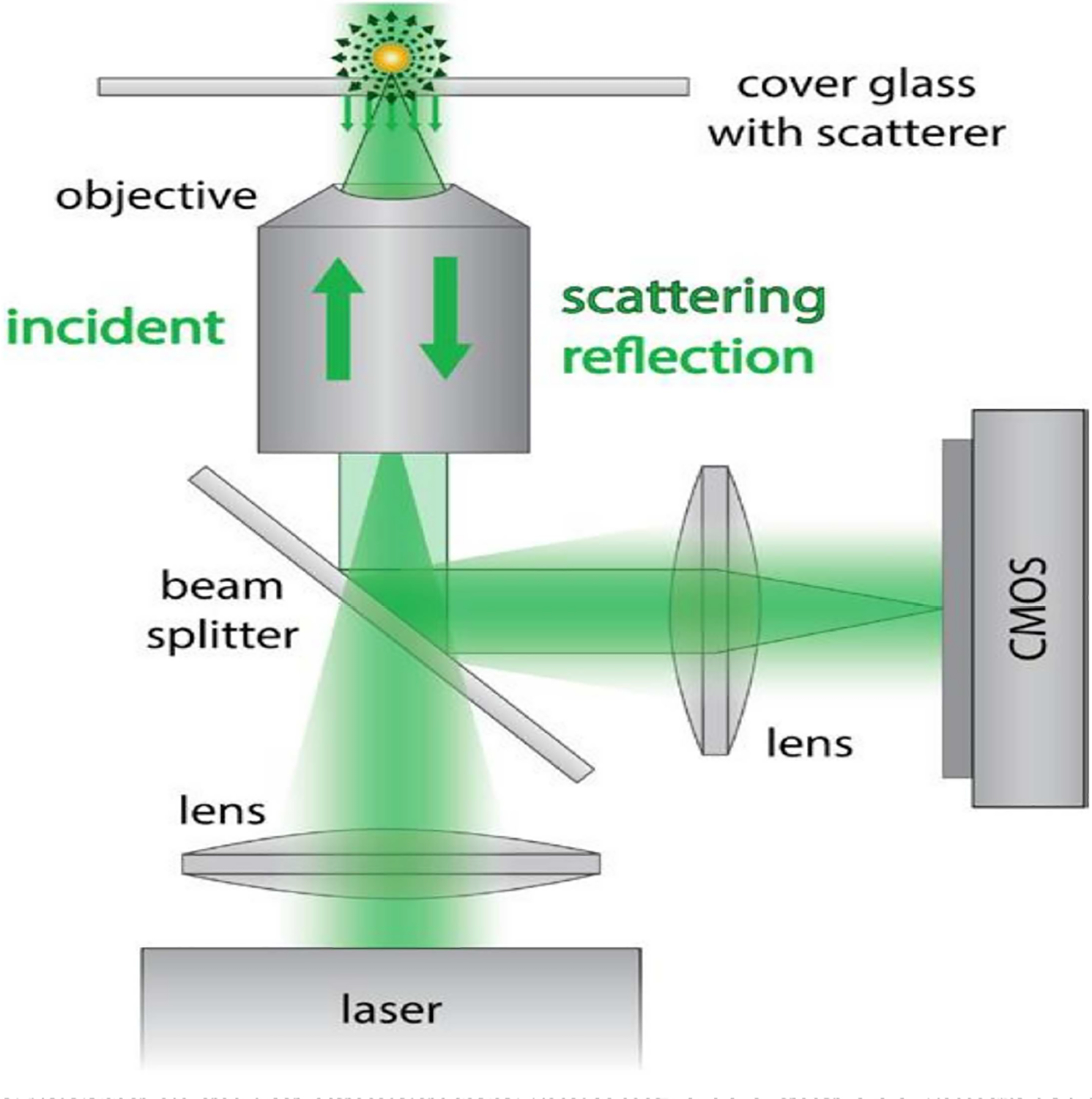
Schematic of the Interferometric scattering microscopy. Laser light is focused on the back focal plane of the high numeral aperture microscope objective. The reference and scattered light from the sample are reflected by a beam splitter and captured by a CMOS camera. Reproduced with permission from reference [163]. Copyright (2016) IOP Publishing.

Interferometric scattering microscopy (iSCAT) is a label free platform capable of detecting, imaging and tracking the movement of single particles [155]. Optical detection of gold nanoparticles smaller than 10 nm in diameter using interferometric approaches was reported by Lindorfs and colleagues in 2004 [156]. This technique relies on the measurement of scattered light from the sample using an optical microscope [156]. Imaging in iSCAT is conducted in a reflective geometry and follows the same concept of widely used techniques such as reflection interference contrast microscopy [157] or interference reflection microscopy [158]. iSCAT achieves improved sensitivity through the use of a coherent light source and optimized detection techniques [159]. In iSCAT, a laser beam (445 nm) illuminates a glass substrate through an imaging objective, and the reflection from the substrate-water interface is used as a reference for interferometric detection [160]. The nanoscale object of interest on the substrate and any inhomogeneities on the surface generate scattering, which is subsequently collected by the objective (Figure 6). Both the reference and scattering are captured by the complementary metal oxide semiconductor (CMOS) camera as planar and converging spherical waves resulting in the interference since these two optical fields are coherent [161]. In addition, combining the polarizing beam splitter with a quarter wave plate ensures that the reflected and scattered lights can be adequately extracted. The light intensity incident on the detector from the scattered and reflected light is given by the following equation:

$$I_{\text{det}}={\left|E_{r}+E_{s}\right|}^{2}={\left|E_{i}\right|}^{2}\left[r^{2}+{\left|s\right|}_{2}-2r\left|s\right|\;\text{sin}\;\phi \right]$$

where *E*_*r*_, *E*_*s*_ and *E*_*i*_ are the reflected, scattered, and incident electric field amplitudes, *s* denotes the scattering amplitude, *r* is the reflectivity of the interface, and *φ* denotes the combination of the reflected light field phase and the scattering phase [162]. For weakly scattering particles, the scattering amplitude quickly approaches zero leaving only the interference term (2*r*|*s*| sin *φ*). The CMOS camera observes essentially uniform illumination across the sample region due to the rapid scanning of the incident light by acousto-optical deflectors at a rate faster than the camera exposure time [155]. The image from the camera is made up of small features visible against the background as a result of weakly scattering particles. Noise reduction is performed by the camera, through the build-up of photoelectrons over time.

A major advantage of the iSCAT technique is that it enables the detection of Rayleigh scattering of individual biomolecules in a straightforward manner without the use of labels. Furthermore, the signals obtained from single molecules show a single distribution of the maximum signal which is directly proportional to the mass of the analyte as compared to single molecule visualization techniques such as those based on plasmonics and or cavity-based resonance where signal can fluctuate between zero and some maximum, thereby making quantification difficult. This technology has been used to track the motion of proteins [155], and to measure the association/dissociation kinetics of biological analytes [161]. Kukura et al. [162] combined single molecule fluorescence microscopy and iSCAT and were able to visualize the location and orientation of single quantum dot-labeled Simian virus 40 (40 nm diameter) particles in real time. iSCAT has also been used to visualize the mechanisms involved in the formation of supported lipid bilayers (SLB) which has been difficult to observe [164]. iSCAT enabled direct observation of single vesicle adsorption and the subsequent transformation into a planar bilayer. The capability of this technique to detect and localize multiple particles down to a diameter of 5 nm in a diffraction-limited spot shows potential for super resolution without a fluorescence-based imaging modality. A drawback to iSCAT is that minimal information can be obtained about the biomolecule beyond its molecular weight, and that nonspecific scattering can result in signals with high background when residual scattering cannot be controlled.

### 3.4. Dark-Field Microscopy

Dark-field microscopy has been utilized for many decades, and its capabilities were explored in the 17th century by Hooke, von Leeuwenhoek and Huygens [165,166]. In 1903, Stedentopf and Zsigmondy developed a different type of dark-field microscopy, called the “ultramicroscope”, which made it possible to visualize gold nanoparticles with diameters smaller than 10 nm [167]. Their microscope enabled them to observe and count single metal nanoparticles in liquid and estimate their size. They also studied the kinetics of particle coagulation and structure of a variety of heterogeneous systems [168]. The capability for studying both hard and soft materials has resulted in widespread use of dark-field microscopy in material science and biology.

There are many different types of dark-field microscopy that have been developed that share common features of detecting scattered light from a planar surface and suppressing featureless samples to render a dark image [169]. Light scattered or emitted from the sample of interest is detected against a dark-background, which can boost the signal to noise ratio of the image and allow the visualization of very small sample features. In conventional dark-field microscopy a light stop blocks the central part of the illumination light, which normally passes through and around the sample in bright field microscopy, thereby only allowing oblique rays to hit the sample [170]. In this arrangement, the light reflected or transmitted by the sample is not collected by the objective and the only light that will be collected is the scattered light from the sample against a dark background. The resolution of the system can be improved by using a high numerical aperture configuration of the objective/condenser lens pair.

Dark-field microscopy can be coupled to an imaging monochromator and a camera in order to measure the Rayleigh scattering spectra from single particles [171]. For nanoparticles made of metals such as gold or silver, the resultant spectrum is dominated by localized surface plasmon resonance (LSPR) whereby an increase in the optical absorption and scattering is noted at a resonant frequency when the oscillation of electrons is confined by the nanoscale size of the particle [172]. The resonance of these nanoparticles is extremely strong and their absorption and scattering is many times greater than that resultant from their geometry alone, making them useful as a non-bleaching label for optical imaging of biomolecules of interest [173].

### 3.5. Photonic Crystal Enhanced Microscopy

Earlier we described several papers utilizing PC-based sensors for single particle detection that used known defects or variations to provide single-location identification of binding events. These imaging techniques use known locations that can be monitored for particle binding events that happen over time. As discussed with the other methods in this section, there are benefits to imaging a large region of interest, which can be done across an entire PC surface by repeated line scanning of to produce a three-dimensional image-stack which can be analyzed for spatially-resolved spectral responses due to particle binding. Chen, et al. described the technique with the goal of providing a label-free imaging method for cellular attachment, detachment, and differentiation [174–176]; however, Zhuo, et al. further demonstrated the ability to use this non-modified one-dimensional PC to provide a spatially-resolved reflectance image of a 50 μm × 50 μm area capable of detecting individual titanium and gold nanoparticles [177].

**Figure 7.**
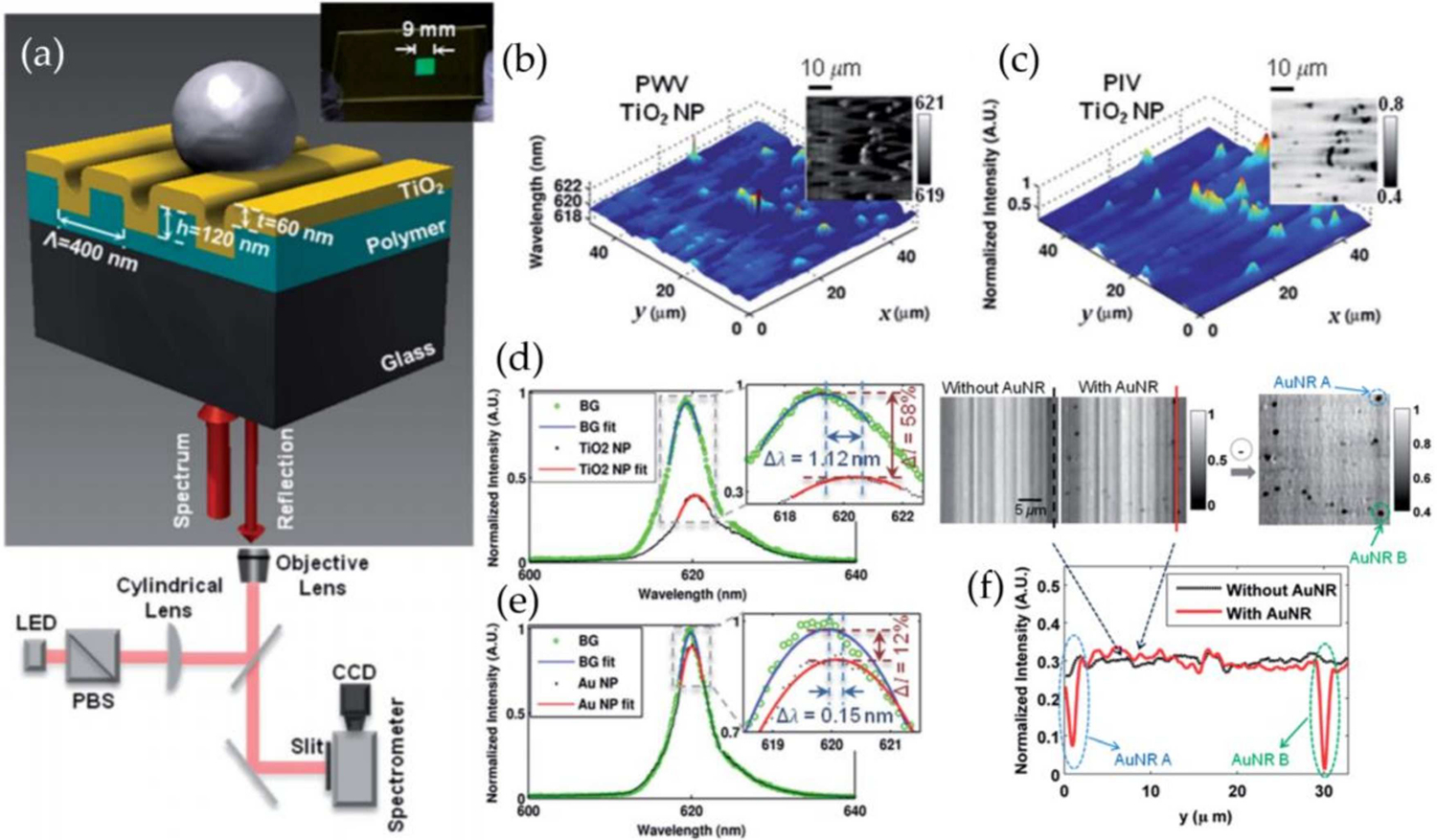
Photonic Crystal Enhanced Microscopy. (**a**) Schematic diagram of a nanoparticle attached to a PC surface. Inset: photo of a PC fabricated on a glass cover slip. (**b**,**c**) Photonic Crystal Enhanced Microscope (PCEM) detection of randomly distributed TiO_2_ nanoparticles showing peak wavelength value (PWV) and peak intensity value (PIV) shift images, with representative spectra shown in (**d**). (**e**) Representative spectra of an Au nanoparticle showing PWV and PIV shifts. (**f**) PIV images of Au nanoparticles randomly distributed with a profile view of a single line scan showing clear spatially resolved PIV dips of two nanoparticles.

The Photonic Crystal Enhanced Microscope (PCEM) is comprised of a fiber-coupled LED collimated and polarized so as to illuminate the PC with light with its electric field vector oriented orthogonal to its grating structure. This illumination is focused to a line on the sensor surface, and the light is reflected to the imaging slit of a spectrometer, thereby providing the resonance spectrum for each point along the illumination line (Figure 7a). By moving the PC sensor with a linear stage, a line-scanned image can be generated, providing a 2D spatial image with complete reflected spectrum at each effective pixel. Zhuo, et al. demonstrated that the resolution of these images is sufficient to identify single-particle attachment via two different alterations to the resonance peak: a bathocromic shift of the peak wavelength value (PWV) resulting from an increase in the effective refractive index of the PC sensor due to particle attachment, as well as a decrease in the peak intensity value (PIV) due to particle outcoupling or absorbance from the waveguide. To demonstrate single binding events, the PC sensor was coated with anti-rabbit IgG, and gold nanorods were coated with rabbit IgG antibodies. Individual particle attachment events were observed and analyzed (Figure 7b–d).

### 3.6. Super Resolution Microscopy with Microspheres (SMON)

**Figure 8.**
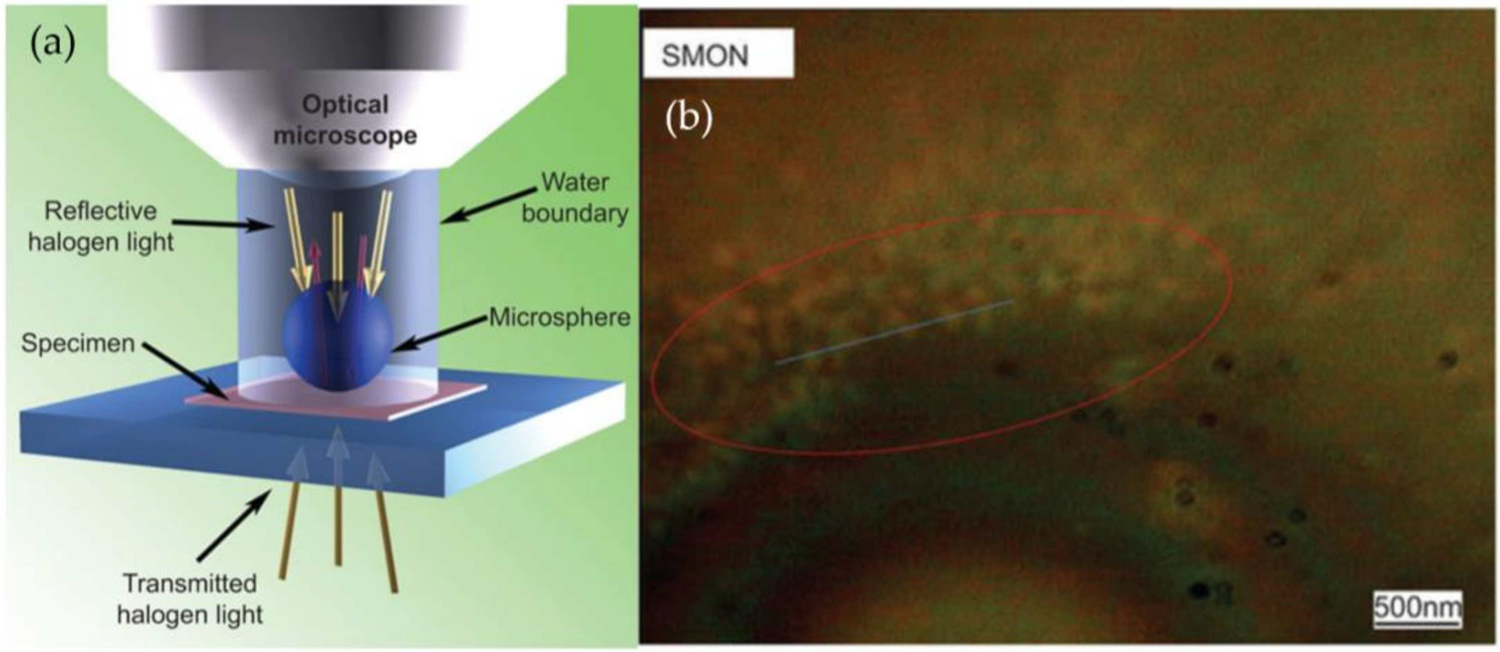
Super Resolution Microscopy with Microspheres (SMON). (**a**) Schematic of SMON instrument showing reflective and transmissive illumination and the location of both specimen and microsphere. (**b**) Representative SMON image showing adenovirus clusters in solution. Reproduced with permission from [182]. Copyright (2013) Springer Nature.

Several demonstrations of white light super-resolution microscopy using microspheres have been recently reported in the literature, and provide a possible opportunity for digital particle detection; however, much of the work in this area has been focused on understanding, modeling, and optimizing the technique [178–181]. To our knowledge, Li, et al. describes the only microsphere-based detection of nanoparticles [182]. Li discusses the mechanism behind this super-resolution microscopy as one based upon the conversion between near-field evanescent waves and far-field propagating waves; briefly, collimated illumination from beneath the sample passes through the sample and into the microsphere where it is focused, and some of that focused light is reflected by the second interface of the microsphere and an aqueous solution (Figure 7a). This light is refocused by the microsphere and produces photonic nanojets. By reversing this optical path, light scattered from the sample can be converted into propagating waves that are collected by a standard microscope objective. It is worth mentioning that other mechanisms for the observed superresolution phenomenon have also been reported [179,180]. Regardless of the fundamental mechanism, Li was able to demonstrate the ability to resolve 75-nm adenovirus particles via this technique (Figure 8b). Though not used to measure individual particles, the possibility exists to use such a super-resolution technique for digital particle detection as many of the other methods discussed.

## 4. Conclusions

This review has described the underlying physical principles, sensor configurations, instrumentation, and applications for nanoparticle-tagged biodetection with the capability for digital resolution. Through the ability to observe the attachment of a single dielectric or metallic nanoparticle on an optical transducer, individual biomolecules or virus particles can yield easily observable signals that can be used to signal their presence and to count them. We have reviewed broad classes of surface plasmon resonant surfaces dielectric resonators, thin film interference effects, and scatting microscopies that share the capability for single-nanoparticle detection limits, and we broadly classified detection methods as those that measure aggregated signals, versus those that can generate images of captured nanoparticle tags.

The technologies described in this review are moving towards approaches that can be applied as life science research tools and in vitro diagnostics assays. Importantly, they can enable direct detection of selectively captured viral particles, and nanoparticle-tagged detection of nucleic acid or protein molecules that serve as biomarkers of disease. Importantly, nanoparticle tags do not require enzymatic amplification and unlike fluorescent reporters, are not subject to the effects of photobleaching, which enables real-time kinetic monitoring of the detection process, with accurate quantification of the analyte. Further, nanoparticle-tagged digital-resolution detection occurs instantaneously when the analyte attaches to the sensor, enabling the collection of dynamic information and the ability to report a result rapidly. We expect, in the near future, that several of the technologies reviewed here will move towards clinical translation and broad adoption in the life science research community. The methods described in this review are expected to become important tools for some of the most demanding challenges in biosensing, in which a very small number of available analytes are present in test samples of limited volumes. These types of applications can include detection of low-abundance nucleic acid (miRNA, mRNA, or circulating tumor DNA) or protein biomarkers within a droplet of serum, detection of a small number of viral particles in a sample of bodily fluid, or studying the proteomics within individual cells. In each of these situations, the ability to digitally count the analytes with a simple detection process will lead to simple workflows, lower costs, and more rapid acquisition of results.
